# Effects of a Novel Dental Gel on Plaque and Gingivitis: A Comparative Study

**DOI:** 10.4172/2161-1122.1000239

**Published:** 2014-05-30

**Authors:** M Dadkhah, NE Chung, J Ajdaharian, C Wink, P Klokkevold, P Wilder-Smith

**Affiliations:** 1 Beckman Laser Institute, University of California, Irvine, California 92612, USA; 2 School of Dental Hygiene, Concorde Career College, Garden Grove, California 92840, USA; 3 School of Dentistry, Section of Periodontics, University of California, Los Angeles, California 90095, USA

**Keywords:** Plaque, Gingival inflammation, Dentifrice, Oral hygiene, Biofilm

## Abstract

**Objectives:**

The goal of this prospective, randomized, controlled, double-blinded study was to evaluate the effects of a novel dental gel on plaque and gingival health. The dental gel was designed to (1) break up and prevent re-accumulation of microbial biofilm, and (2) inhibit metal mediated inflammation.

**Materials and Methods:**

Twenty-five subjects with moderate gingival inflammation (Löe and Silness Gingival Index ≥2) and pocket depths <4 were randomly assigned to brush twice daily for 21 days with the test or the control dental gel. On Days 0, 7, 14 and 21, plaque levels (Quigley-Hein, Turesky Modification Plaque Index), gingival inflammation (Löe and Silness Gingival Index) and gingival bleeding (modified Sulcus Bleeding Index) were determined by one blinded, investigator using a pressure sensitive probe.

**Results:**

After 3 weeks, all 3 clinical indices were significantly improved in both groups (P<0.05) and significantly lower in the test group (P<0.05).

**Conclusion:**

The novel dental gel formulation was provided effective plaque control and reduced gingival inflammation.

**Clinical Relevance:**

A novel dentifrice formulation may be an effective tool for plaque removal and maintaining gingival health.

## Introduction

Studies in the USA and the UK suggest that some degree of gingivitis affects 50–90% of the adult population [1]. A recent study evaluating data from the 2009 and 2010 National Health and Nutrition Examination Survey (NHANES) found that 47% of adults had periodontitis [2]. Oral biofilm is the primary etiological factor in the initiation of gingival inflammation and subsequent destruction of periodontal tissues [1]. Effective oral hygiene which eliminates microbial plaque leads to resolution of gingival inflammation [3]. Conversely, lapses in plaque control result in recurrence of inflammation [4].

Despite its essential role in the prevention of gingivitis and periodontitis, mechanical plaque control is not adequately practiced by most individuals. A survey conducted in the United Kingdom concluded that one-third of teeth in 72% of all dentate adults had visible plaque [5]. Achieving effective plaque control on a regular basis is challenging: the majority of the normal adult population does not achieve the levels of plaque control needed to maintain gingival and periodontal health [6]. A review of the effectiveness of self-performed mechanical plaque removal in adults with gingivitis concluded that the quality of conventional mechanical plaque control was not effective in reducing gingivitis [7]. Professional cleaning is available to only a small proportion of the world's population; therefore improvements in the approach to plaque control are needed to address widespread dental disease. Consequently, a multitude of novel formulations are under investigation for their ability to remove oral biofilm and to discourage its re-accumulation [6,8,9].

Various active agents have been investigated for their ability to augment mechanical plaque control measures. The use of chlorhexidine preparations can be beneficial; however their side effects such as staining and alterations in taste sensations preclude long-term usage [10]. Dentifrices containing triclosan co-polymer can improve oral biofilm control, however in some studies excellent short-term benefits were offset by lack of evidence for any long-term benefits [11]. In addition there is an ongoing debate about potentially undesirable effects including allergic sensitization [12], disruption of endocrine function [13], and antibiotic resistance [14]. Thus the search continues for a better adjunct agent to mechanical plaque control.

Cations such as calcium and iron are essential to microbial adherence, biofilm formation, and bacterial growth. Because the metal-binding chelator edathamil has the capability to inhibit biofilm, there exists the potential for employing it to disrupt surface adherence of dental plaque and inhibit biofilm production [15]. Moreover, calcium and iron also play critical roles in the inflammatory process [16], so that the use of a metal-binding agent such as edathamil may have an additional beneficial effect on mitigating inflammation. Recent studies have determined that metal chelation inhibits the formation of cytotoxic 4-Hydroxynonenal (HNE) and the initiation of apoptotic/inflammatory events [17,18].

The goal of this prospective, randomized, controlled, double-blinded study was to determine the effects of a novel dentifrice on plaque and gingival health. The dental gel tested in this study contains a 2.6% proprietary formulation of activated edathamil to target biofilm disruption and discourage biofilm re-formation on the tooth surface. It was postulated that the novel dental gel will favorably affect oral hygiene and gingival health through (1) improved plaque removal, (2) reduced plaque accumulation and (3) local anti-inflammatory effects.

## Materials and Methods

This project was performed in full compliance with University of California at Irvine IRB-approved protocol #2002-2805. Written informed consent was obtained from all participants in this study.

### Subjects

Twenty-five subjects in good general health ranging in age from 19-31 years old (mean age of 23 years) with mild to moderate gingival inflammation (Löe and Silness Gingival Index ≥2) [19,20] and pocket depths ≤4 were enrolled in this prospective, randomized, controlled, double-blinded study. They had received professional prophylaxis 4-7 weeks before enrollment in this study. An interval of 4-7 weeks was chosen to allow time for the gingiva to heal after prophylaxis. Subjects were students at the University of California, Irvine, recruited by email through the UCI subject recruitment service. Sample size was calculated using a power of 0.90 and the differences in the mean values and standard deviations obtained from a prior pilot study. This resulted in a sample size per arm of 9. Thirteen (13) subjects were female and 11 were male; 11 were Caucasian, 11 Asian and 2 African-American. Subjects were screened to exclude persons with any known history of allergy to personal care/consumer products or their ingredients, and any ingredients in the test product. Other exclusion criteria included any medical condition which requires pre-medication prior to dental visits/procedures, any diseases of the soft or hard oral tissues, use of antibiotics one month prior to or during this study, pregnancy or lactation, as well as immune compromised individuals (HIV, AIDS, immuno-suppressive drug therapy). The participants were randomized in groups of 6, with 3 in each arm per group to ensure an evenly divided study in case subject accrual did not achieve its targeted goal of 25.

### Clinical Protocol

Subjects were randomly assigned to brush twice daily for 21 days with either the test dental gel (2.6% Livionex^R^ Dental Gel, Los Gatos, CA), or the control gel (Colgate Total^R^, Colgate-Palmolive, Piscataway N.J.). Colgate Total^R^ was used because it its primary active component, triclosan co-polymer is a substance widely used for oral biofilm control, with some studies showing excellent short-term benefits [11]. A standard Oral B ProFlex^R^ toothbrush was provided to each volunteer and subjects were trained in standard sulcular brushing technique. Use of any other oral hygiene measures was not permitted, included mouthwashes and chewing gum. At each visit this information was repeated to the subjects and a written information sheet was also sent home with them after each visit. Subjects brushed their teeth two hours prior to each visit and refrained from eating from that time onwards until after their visit. The two-hour interval after brushing was scheduled for several reasons: (1) to avoid mis-charting gingival health due to immediate and direct tooth-brushing initiated bleeding, (2) because it is difficult to motivate subjects to spend the first part of their day with un-brushed teeth and concerns about halitosis. Plaque levels (Turesky Modification of Quigley-Hein Index [21]) (P.I.), gingival inflammation (Löe and Silness Gingival Index [20]) (G.I.), and sulcus bleeding (mSBI) [22]), were recorded. A standardized pressure sensitive probe (Florida Probe) with 20 g probing force was used to measure probing depth. Volunteers were photographed and evaluated on Days 0, 7, 14, and 21 by the same blinded, pre-calibrated investigator. The investigator was pre-calibrated to 95% accuracy on 50 periodontal patients by an experienced periodontist with over 30 years of experience in periodontal diagnosis. All investigators and subjects were blinded to the dental gel identity by the use of identical toothpaste tubes labeled only with a coded number. Only the study nurse manager had access to the key for the sample codes. Subjects were monitored and questioned regarding any adverse effects at each visit and also provided with a direct telephone number to contact in case of any adverse effects.

Compliance was measured by a weekly take-home form on which subjects recorded time and duration of each tooth brushing. Additionally, they brought their tube of dental gel with them to each appointment and weekly dentifrice usage was determined by weighing the tubes and computing tube weight loss between visits.

### Measured Indices

### Quigley-Hein Plaque Index

Plaque was scored according to the Turesky modification of the Quigley-Hein Plaque Index. A score of 0 to 5 is assigned to each facial and lingual non-restored surface of all the teeth according to the following criteria:

0 = No plaque.
1 = Separate flecks of plaque at the cervical margin.
2 = A thin, continuous band of plaque (up to 1 mm) at the cervical margin.
3 = A band of plaque wider than 1 mm, but covering less than 1/3 of the side of the crown of the tooth.
4 = Plaque covering at least 1/3, but less than 2/3 of the side of the crown of the tooth.
5 = Plaque covering 2/3 or more of the side of the crown of the tooth.

### Löe-Silness Gingival Index

Each tooth was divided into two surfaces, facial and lingual. Those teeth with cervical restorations or prosthetic crowns were excluded from the scoring procedure. The gingiva adjacent to each tooth surface was scored as follows:

0 = Absence of inflammation.
1 = Mild inflammation: slight change in color and little change in texture.
2 = Moderate inflammation: moderate glazing, redness, edema, and hypertrophy.
3 = Severe inflammation: marked redness and hypertrophy. Tendency for spontaneous bleeding

### Modified Sulcus Bleeding Index

A score of 0 to 3 was assigned to each facial and lingual non-restored surface of all the teeth according to the following criteria:

0 = No bleeding when periodontal probe is passed along the gingival margin
1 = Isolated bleeding spots visible
2 = Blood forms a confluent red line on the gingival margin
3 = Heavy or profuse bleeding

## Results

**Figure 1:**
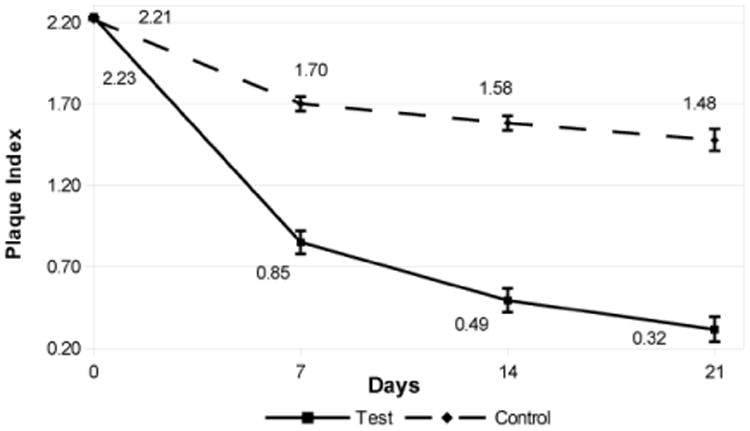
Plaque Index at Baseline, Day 7, Day 14, and Day 21. The error bars indicate the standard error of the mean (σ/√n)

**Figure 2:**
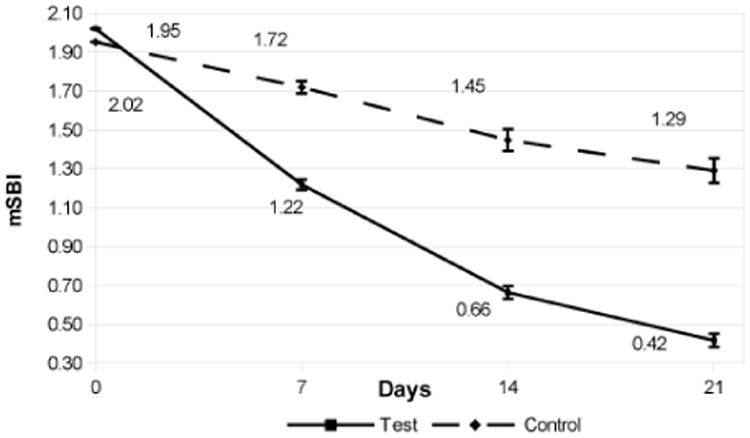
Modified Sulcus Bleeding Index (mSBI) at Baseline, Day 7, Day 14, and Day 21. The error bars indicate the standard error of the mean (σ/√n)

**Figure 3:**
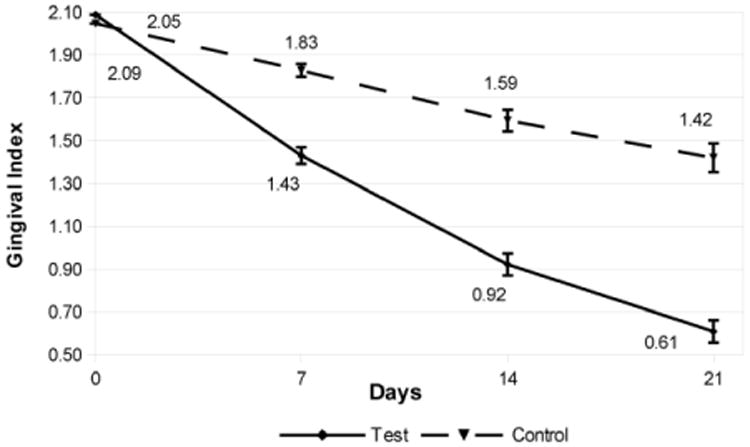
Gingival Index at Baseline, Day 7, Day 14, and Day 21. The error bars indicate the standard error of the mean (σ/√n)

The Indices at baseline, days 7, 14, and 21 are shown in Figures 1-3. The comparative data is summarized in Table 1. Briefly, clinical indices at study outset were comparable in the 2 groups. At Baseline, P.I. averaged 2.2 in both groups; mean G.I. measured 2.1 in the test group vs. 2.0 in the control group, and mSBI averaged 2.0 in both groups. Over the next 3 weeks, P.I., G.I. and mSBI diminished progressively, showing a significant improvement in each group at 3 weeks vs. the original value (P<0.05). The test group showed significantly lower values for all indices vs. the control group at all intervals over the 3 week period.

The P.I. showed an 86% reduction in plaque in the test dental gel (Livionex) compared to a 33% reduction for the control dental gel (Colgate Total^R^). The mSBI showed a 79% reduction in bleeding with the test dental gel compared to a 34% reduction for the control dental gel. The G.I. showed a 71% reduction in gingival inflammation in the test dental gel compared to a 31% reduction for the control dental gel.

## Discussion

Using conventional clinical indices for plaque and gingival health, a significant reduction in plaque levels and gingival inflammation was apparent in both treatment groups after 3 weeks. This observation was not unexpected. The subjects enrolled in this study all initially evidenced poor oral hygiene; most likely they were strongly motivated to improve their oral hygiene by the prospect of weekly oral exams, and the knowledge that they were enrolled in a study to identify the effects of a new dental gel. These subjects were young and in excellent general health, and their oral health improved quickly and considerably with improved oral hygiene practices. Similar effects of improvements in oral hygiene during plaque-removal studies have been described in many other studies [23-25].

The lower plaque indices recorded in the group using the test gel were clearly evident on the first evaluation day of the study, Day 7, and further progressive Plaque Index reduction was observed on Days 14 and 21 of the study. These findings support the initial hypothesis that the novel dental gel formulation may provide improved plaque control. Moreover, the progressive reduction in Plaque Index over the entire 21 day duration of the study, whilst not conclusive, may potentially be an indicator of diminished plaque re-formation on the teeth over time. Whilst some previous studies have demonstrated effective plaque inhibition by edathamil [15,26], one of the active agents in the novel toothpaste formulation, other publications describe only a limited anti-plaque effect of the chelating agent, attributed to its lack of penetration into biofilm [27]. A carrier and permeability enhancer (the formulation contains only FDA GRAS (Generally Regarded as Safe) and natural ingredients) was incorporated into the test dental gel formulation to promote edathamil penetration into the dental plaque and enhance its anti-plaque efficacy [28]. This plaque-disruptive effect was observed in a recent in situ high-resolution imaging study, where, at the same sites on specific teeth, considerably smaller amounts of biofilm were consistently recorded when subjects used the test toothpaste vs after use of over-the-counter toothpaste (Lasers in Surgery and Medicine, in press). Moreover, the residual plaque was appeared less dense when subjects used the test toothpaste. The greater improvement in gingival inflammation achieved in the subjects using the test gel may be attributed either to the better plaque removal observed in this group, or to the anti-inflammatory effect of the gel formulation [17,18,29], or to a combination of the 2 factors.

Many studies document reduced plaque and gingivitis in subjects using toothpaste containing the active ingredient of Colgate TotalR, Triclosan co-polymer [30]. This is a broad-spectrum antibacterial agent effective against both gram-positive and gram-negative bacteria. Given the very different mechanisms of action of the 2 dentifrices tested in this study, further studies using more sophisticated techniques for documenting plaque and inflammatory status over longer evaluation periods than the 3 weeks used in this research are necessary to gain a better and more nuanced understanding of the merits of each toothpaste.

## Conclusion

In this clinical study a novel dental gel achieved excellent plaque control and reduced gingival inflammation over a period of three weeks.

## Figures and Tables

**Table 1:** Change in Clinical indices (SD) for Control and Test groups from Baseline on Day 7, Day 14 and Day 21. n=13 for the Test Group, and n=12 for the Control Group

	Day	Control Difference from Baseline (SD)	Test Difference from Baseline (SD)	df	T-stat	P-Value
Plaque Index (0-5)	7	-0.51 (0.34)	-1.38 (0.57)	19	-4.63	0.00018
	14	-0.63 (0.34)	-1.74 (0.59)	19	-5.77	0.00002
	21	-0.74 (0.52)	-1.92 (0.62)	22	-5.19	0.00003
Modified Sulcus Bleeding Index (0-3)	7	-0.23 (0.22)	-0.80 (0.19)	22	-6.94	0.000001
	14	-0.50 (0.38)	-1.36 (0.24)	18	-6.62	0.000003
	21	-0.66 (0.43)	-1.60 (0.25)	17	-6.67	0.000004
Gingival Index (0-3)	7	-0.22 (0.21)	-0.66 (0.29)	21	-4.34	0.00028
	14	-0.45 (0.35)	-1.17 (0.39)	23	-4.82	0.00008
	21	-0.63 (0.48)	-1.48 (0.40)	21	-4.84	0.00009
